# Off-label GLP-1 receptor agonist and tirzepatide use for weight loss: patient safety and regulatory oversight

**DOI:** 10.1097/MS9.0000000000005191

**Published:** 2026-06-17

**Authors:** Areesha Maqsood, Abdul Rafay Khan, Syeda Ilsa Aaqil, Asad Ali Ahmed Cheema, Maher Ali, Jerril Jacob, Muhammad Naeem, Ahmed Kamal Siddiqi

**Affiliations:** a Department of Medicine, Aga Khan University, Karachi, Pakistan; b Department of Medicine, Ziauddin University, Karachi, Pakistan; c Department of Medicine, Jinnah Sindh Medical University, Karachi, Pakistan; d Department of Medicine, International School of Medicine, International University of Kyrgyzstan, Bishkek, Kyrgyzstan; e Department of Medicine, International School of Medicine, International University of Kyrgyzstan, Bishkek, Kyrgyzstan; f Department of Internal Medicine, Rutgers New Jersey Medical School, Newark, NJ, USA; g Division of Cardiothoracic Radiology, Department of Radiology, Mayo Clinic, Phoenix, AZ, USA; h Division of Cardiothoracic Imaging, Department of Radiology and Imaging Sciences, Emory University, Atlanta, GA, USA

Both semaglutide, a GLP-1 receptor agonist, and tirzepatide, a dual GIP/GLP-1 receptor agonist, are changing the way we treat type 2 diabetes mellitus, obesity, as well as an increasing number of other cardiometabolic diseases. Major adverse cardiovascular events (MACE) were reduced by 20% in overweight/obese individuals with pre-existing cardiovascular disease without diabetes treated with semaglutide 2.4 mg compared to placebo (6.5% vs. 8.0%; absolute risk reduction [ARR] 1.5%, number needed to treat [NNT] ≥ 67 over ∼4 years)[1]. Semaglutide also improved renal outcomes, reducing major kidney disease events in patients with type 2 diabetes and chronic kidney disease by 24% [hazard ratio (HR) 0.76; 95% confidence interval (CI) 0.66–0.88; *P* = 0.0003][2]. Additionally, the ESSENCE study demonstrated that semaglutide 2.4 mg provided MASH resolution without worsening fibrosis in 62.9% of participants at week 72, resulting in FDA accelerated approval in August 2025 (awaiting confirmation results from the ongoing ESSENCE Part 2 Study due in 2029)[3]. There is emerging evidence supporting benefits in treating substance use disorder as well, through modulating central reward pathways. It is clear that there is real potential for this class of drugs; however, the increased use of these agents as off-label treatments for cosmetic weight loss, especially when used inappropriately under no clinical supervision, presents many serious concerns regarding both ethics and public health, which need to be addressed immediately.

Unregulated compounded product use (as opposed to regulated pharmaceutical product use) has created a significant increase in the risk to patient safety. The FDA has repeatedly expressed concern about products that can legally circumvent regulatory review for their safety, efficacy, and quality. By 31 July 2025, more than 1150 adverse event reports had been filed by patients using compounded semaglutide and tirzepatide in combination – a number that undoubtedly represents a conservative estimate due to the lack of mandatory reporting requirements for all state-licensed compounders. By 31 December 2024, at least 17 of those cases were deaths reported to the FAERS (according to Senator Banks, quoting FAERS, NAM, August 2025)^[^4,5^]^. Dosing errors have proven to be especially worrisome, with patient misadministration due to unfamiliarity with multi-dose vials leading to five to twenty times the prescribed dose, causing adverse reactions ranging from nausea, vomiting, and dehydration to acute pancreatitis, gallstones, and syncope[4]. In an analysis published by the Brookings Institution in 2025, Chinese manufacturers representing about 20% of semaglutide compounds imported for compounding had never been inspected by the FDA, whereas companies responsible for more than 44% of imports had been found guilty of violations during inspection[5]. Unsupervised patients might also take these drugs when there are definitive contraindications present, such as a family history of medullary thyroid carcinoma or multiple endocrine neoplasia type 2, which can only be determined with the aid of a physician.

Despite being well indicated for use, GLP-1RAs have a distinct adverse effect profile, which is commonly underestimated in society. Delayed gastric emptying has proven to be one of their most relevant perioperative risks: case reports of pulmonary aspiration in fasted individuals under anesthesia, while taking semaglutide, prompted the FDA to amend its labeling despite standard preoperative fasting and not knowing how long to stop using semaglutide prior to surgery. In terms of pancreatitis, we must remember the need to properly contextualize these data. A recently updated meta-analysis from 2024, which considered 21 placebo-controlled randomized trials of semaglutide use (totaling 34 721 patients), failed to show any increased risk of acute pancreatitis compared to placebo (odd ratio [OR] 0.7; 95% CI 0.5–1.2; *I*^2^ = 0%)[6]. An observational study in 2023 from the Journal of the American Medical Association, examining adults without diabetes and taking GLP-1RAs for obesity treatment, showed a significantly increased pancreatitis risk compared to patients taking naltrexone-bupropion (HR 9.09; 95% CI 1.25–66.00). It is important to note that obesity and type 2 diabetes are independent pancreatitis risks[7]. A 2025 PRISMA-based meta-analysis of 62 randomized clinical trials using all GLP-1 RA drugs (*N* = 66 232) identified a significant class effect with regards to an increased risk of acute pancreatitis (risk ratio 1.44, 95% CI 1.09–1.89, *P* = 0.009), which was reduced to statistical insignificance following adjustment for confounders based on co-medication[8]. Semaglutide’s link with nonarteritic anterior ischemic optic neuropathy (NAION) has been officially established. As of June 2025, the PRAC of the European Medicines Agency confirmed NAION to be a very rare side effect (up to 1 in 10 000 individuals) and updated the relevant European labeling, with the WHO publishing a similar global safety warning during the same period[9]. Patients experiencing sudden painless vision loss during treatment should stop using semaglutide and have themselves examined by a specialist. Data from the STEP 1 trial extension show that 1 year following discontinuation of semaglutide, patients regained a mean of 11.6 out of the 17.3 percentage points of weight lost while on the drug (about two-thirds of the previous weight loss). Improvements in cardiometabolic parameters partially revert back to, but do not fall below, their pretreatment levels[10]. Body-composition evidence from semaglutide clinical trials indicates that weight reduction is driven predominantly by fat-mass loss, while measured lean-mass changes are smaller and should be interpreted cautiously[11]. Therefore, weight regain after discontinuation should be discussed within a broader clinical framework that includes nutrition, resistance training, functional monitoring, and avoidance of unsupervised or cosmetic use. Regarding the psychological–social aspects, a 2023 FAERS analysis uncovered a four-fold higher proportional reporting rate (PRR) for semaglutide misuse compared to other GLP-1 receptor agonists (PRR ≈ 4.05 for drug abuse and withdrawal syndrome), with no statistical differences between semaglutide and the Schedule IV weight-loss compound phentermine-topiramate (phentermine being the sympathomimetic; topiramate the anticonvulsant)[12]. Social media promotion has been especially dangerous amongst the youth population: clinicians and advocacy groups have reported cases of adolescents or young adults with active or dormant anorexia nervosa acquiring semaglutide via online questionnaire manipulation, in some cases eliciting recurrent restrictive and obsessive-compulsive symptoms. A 2024 systematic review demonstrated the absence of any randomized trial evidence in patients with anorexia or bulimia nervosa and found that GLP-1RAs can worsen eating disorder pathology in predisposed subjects[13].

The portrayal of GLP-1 treatment as an easy fix rather than a form of comprehensive obesity treatment is even more dangerous. Metabolic bariatric surgery has proven to produce even more effective results when it comes to achieving weight loss, inducing complete remission of type 2 diabetes, and decreasing obesity-related mortality among individuals suffering from severe obesity (*n* = 174 772; HR of all-cause mortality decreased by 49.2%; median life expectancy (LE) increased by 6.1 years in comparison with the usual standard of care)[14]. For instance, the SURMOUNT-5 trial has confirmed that, in spite of significant improvements in the efficacy of pharmacological treatments (e.g., the 15-mg dose of tirzepatide showed 20.2% mean weight loss at 72 weeks), the mortality benefits of bariatric surgery, as well as its superiority to pharmacology with regard to achieving diabetes remission, are currently unmatched[15]. This distinction is clinically important because lean-mass changes during GLP-1RA therapy should not be interpreted in isolation; rather, they require assessment alongside measurement modality, muscle function, nutritional adequacy, and resistance-training support[16]. The perception of GLP-1RAs and GIP/GLP-1RAs as a way of quickly solving one’s appearance issues may cause patients to avoid undergoing comprehensive testing of their condition, which includes diet, mental health, and the use of surgery. Moreover, high discontinuation rates (within a year) due to high costs, side effects, and unrealistic expectations may promote medication dependence.

Indeed, a comprehensive strategy is necessary. Enforcement actions against unauthorized formulations and off-label compounding are required. Education for clinicians should emphasize pre-prescription screening of eating disorder history using the SCOFF questionnaire and the EDE-Q questionnaire, both brief and suitable for routine use in a clinical setting. The SCOFF questionnaire may have greater sensitivity in specialized eating-disorder settings than in community settings; therefore, a positive screening result should prompt referral for specialist evaluation rather than serve as an absolute exclusion criterion. However, at present, no existing national guidelines require the use of either test prior to the prescription of GLP-1 receptor agonists[13]. Information directed to patients should emphasize indications, the newly labeled risk of NAION confirmed in June 2025[13], and risks of unauthorized self-medication, including unknown risks associated with unauthorized API procurement^[^4,5^]^. Longitudinal studies regarding the patterns of misuse, neurobiology behind appetite-suppressing and reward-modulating effects of GLP-1 receptor agonists, and withdrawal approaches, including gradual dose reduction, which requires further randomized evaluation, are highly desirable to facilitate evidence-based guidelines for physicians. GLP-1 receptor agonists are indeed a revolutionary breakthrough in the management of metabolic disorders; such improper use must not detract from their benefits.

## Data Availability

Not applicable. This editorial does not involve new data generation or analysis. All information discussed is derived from publicly available, previously published sources cited within the manuscript.

## References

[1] Lincoff AM Brown-Frandsen K Colhoun HM. Semaglutide and cardiovascular outcomes in obesity without diabetes. N Engl J Med 2023;389:2221–32. 37952131 10.1056/NEJMoa2307563

[2] Perkovic V Tuttle KR Rossing P. Effects of semaglutide on chronic kidney disease in patients with type 2 diabetes. N Engl J Med 2024;391:109–21. 38785209 10.1056/NEJMoa2403347

[3] Sanyal AJ Newsome PN Kliers I. ESSENCE Study Group. Phase 3 trial of semaglutide in metabolic dysfunction-associated steatohepatitis. N Engl J Med 2025;392:2089–101. 40305708 10.1056/NEJMoa2413258

[4] U.S. Food and Drug Administration. FDA’s concerns with unapproved GLP-1 drugs used for weight loss. Updated February; 2026. Accessed 10 May 2026. https://www.fda.gov/drugs/postmarket-drug-safety-information-patients-and-providers/fdas-concerns-unapproved-glp-1-drugs-used-weight-loss

[5] National Alliance of Manufacturers. Compounded drugs threaten patient safety; 2025. Accessed 10 May 2026. https://nam.org/nam-compounded-drugs-threaten-patient-safety-34515 —Contains: (a) Senator Banks quoting FAERS data as of 31 December 2024 (17 deaths in ≥900 adverse events); (b) summary of Brookings Institution April 2025 API sourcing analysis of Chinese semaglutide manufacturers. These represent two distinct primary data sources cited within this reference.

[6] Masson W Lobo M Barbagelata L. Acute pancreatitis due to different semaglutide regimens: an updated meta-analysis. Endocrinol Diabetes Nutr (Engl Ed) 2024;71:124–32. 38555109 10.1016/j.endien.2024.03.012

[7] Sodhi M Rezaeianzadeh R Kezouh A. Risk of gastrointestinal adverse events associated with glucagon-like peptide-1 receptor agonists for weight loss. Jama 2023;330:1795–97. 37796527 10.1001/jama.2023.19574 PMC10557026

[8] Wen J Nadora D Bernstein E. Evaluating the rates of pancreatitis and pancreatic cancer among GLP-1 receptor agonists: a systematic review and meta-analysis of randomised controlled trials. Endocrinol Diabetes Metab 2025:8:e70113. 40988099 10.1002/edm2.70113 PMC12457091

[9] European Medicines Agency. PRAC Concludes NAION Is a Very Rare Side Effect of Semaglutide (Ozempic, Rybelsus, Wegovy). EMA press release; 2025. See also: World Health Organization. Safety alert: semaglutide medicines and risk of NAION, 27 June 2025.

[10] Wilding JPH Batterham RL Davies M. Weight regain and cardiometabolic effects after withdrawal of semaglutide: the STEP 1 trial extension. Diabetes Obes Metab 2022;24:1553–64. 35441470 10.1111/dom.14725 PMC9542252

[11] Wilding JPH Batterham RL Calanna S. Once-weekly semaglutide in adults with overweight or obesity. N Engl J Med 2021;384:989–1002. 33567185 10.1056/NEJMoa2032183

[12] Chiappini S Vickers-smith R Harris D. Is there a risk for semaglutide misuse? Focus on the FDA Adverse Events Reporting System (FAERS) pharmacovigilance dataset. Pharmaceuticals 2023;16:994. 37513906 10.3390/ph16070994 PMC10384093

[13] Bartel S McElroy SL Levangie D. Use of glucagon-like peptide-1 receptor agonists in eating disorder populations. Int J Eat Disord 2024;57:286–93. 38135891 10.1002/eat.24109

[14] Syn NL Cummings DE Wang LZ. Association of metabolic-bariatric surgery with long-term survival in adults with and without diabetes: a one-stage meta-analysis of matched cohort and randomised controlled studies with 174 772 participants. Lancet 2021;397:1830–41. 33965067 10.1016/S0140-6736(21)00591-2

[15] Aronne LJ Horn DB le Roux CW. SURMOUNT-5 Trial Investigators. Tirzepatide as compared with semaglutide for the treatment of obesity. N Engl J Med 2025;393:26–36. 40353578 10.1056/NEJMoa2416394

[16] Arshad MS Ali M Cheema AAA. Glucagon-like peptide-1 receptor agonists and muscle: interpreting lean mass changes in clinical care. Global Cardiology 2026;4:49–54.

